# Neuronal morphology enhances robustness to perturbations of channel densities

**DOI:** 10.1073/pnas.2219049120

**Published:** 2023-02-14

**Authors:** Yunliang Zang, Eve Marder

**Affiliations:** ^a^Volen Center, Brandeis University, Waltham, MA 02454; ^b^Department of Biology, Brandeis University, Waltham, MA 02454

**Keywords:** compartmental models, ion channels, individual variability, neuronal resilience, parameter space

## Abstract

Biological neurons show significant cell-to-cell variability but have the striking ability to maintain their key firing properties in the face of unpredictable perturbations and stochastic noise. Using a population of multi-compartment models consisting of soma, neurites, and axon for the lateral pyloric neuron in the crab stomatogastric ganglion, we explore how rebound bursting is preserved when the 14 channel conductances in each model are all randomly varied. The coupling between the axon and other compartments is critical for the ability of the axon to spike during bursts and consequently determines the set of successful solutions. When the coupling deviates from a biologically realistic range, the neuronal tolerance of conductance variations is lessened. Thus, the gross morphological features of these neurons enhance their robustness to perturbations of channel densities and expand the space of individual variability that can maintain a desired output pattern.

## Significance

Although neurons show cell-to-cell variability, they can maintain their properties in the face of diverse perturbations. We use a population of compartmental neuron models to reveal that the coupling between neuronal structures is critical for neuronal properties and their robustness. The biological coupling between the axon and other compartments makes neurons resilient to ion channel conductance variation. This suggests a biological strategy that neurons can use to expand the parameter space of cell-to-cell variability that maintains a desired output pattern.

It is now well-established that multiple sets of maximal conductances of ion channels can produce very similar neuronal activity patterns in both individual neurons and circuits (1–10). Nonetheless, it is also clear that models of desired intrinsic properties are relatively rarely found by randomly sampling within a space of parameters (5, 6, 11). Thus, it is likely that biological neurons use homeostatic and other developmental mechanisms to successfully find rare sets of values of ion channel conductances that give rise to specific patterns of intrinsic excitability (12–16).

Although single-compartment, isopotential, conductance-based models have been enormously useful in understanding the relationships among ion channels that give rise to electrical excitability, most biological neurons have complex structures, with differential distributions of channels in the membranes of different neuronal regions. While there are specific examples of conductance-based models of complex neurons (17–22), these models have often been finely tuned to achieve their desired activity patterns. Thus, it is less clear in these more complex models the extent to which complex structures aid or diminish the likelihood of finding vastly different degenerate sets of conductance values that support characteristic activity patterns.

In this study, we exploit a previously developed multi-compartment model database of the lateral pyloric (LP) neuron of the crustacean stomatogastric ganglion (STG) to look at the influence of LP neuron structure on its robustness to perturbations of its channel densities. This is partly motivated by recent studies that show that the fine details of the neuropilar projections of the LP neuron have relatively little impact on how slow synaptic events are processed because of the large length constants of the major neurites (23–25). In themulti-compartment models we study here (soma-primary neurite, axon, two neurite compartments), only the axon compartment has voltage-gated Na^+^ channels (5). To create this model database, Taylor et al. (5) generated ~600,000 randomly selected sets of conductance densities and then sorted them to find ~1,300 models with the desired firing patterns matching the biological properties of the LP neuron. Specifically, when isolated, the biological LP neuron fires tonically, but shows robust rebound bursts in response to inhibitory inputs from its presynaptic partners. These rebound bursts are important in ensuring that the LP neuron fires at an appropriate time during the triphasic pyloric rhythm (26).

The successful ~1,300 models vary considerably in their maximal conductances and are examples of degenerate or multiple solutions that produce a characteristic behavior. In this paper, we look at the effect of neuronal structure on the robustness of this population of LP neuron models in response to perturbations of conductance densities.

## Results

**Fig. 1.**
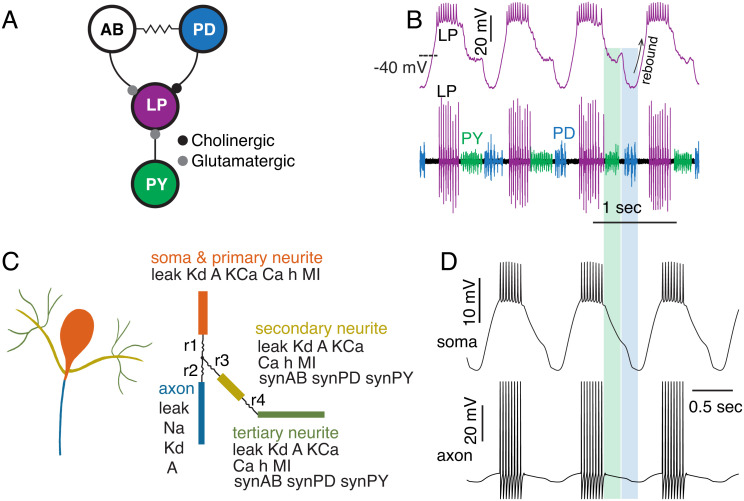
LP neuron multi-compartment model structure and spike properties. (*A*) Schematic of the chemical synaptic inhibition that a LP neuron receives. (*B*) Intracellular recordings of a LP neuron (*Top*) and an extracellular recording at the lvn (*Bottom*, spikes of LP, PY, and PD neurons are color coded, data from Sonal Kedia (27)). (*C*) Schematic of the LP neuron morphology (*Left*) and the model structure (*Right*). Four compartments correspond to axon, soma-primary neurite, secondary neurite, and tertiary neurites with ion channels and synapses distributed in each compartment. (*D*) Example somatic (*Top*) and axonal (*Bottom*) spike waveforms in a model. The green and blue shadings illustrate the timing of the inhibition from PY neurons and PD neurons (also AB neuron).

Fig. 1*A* shows a diagram of the chemical synaptic inputs to the LP neuron. The top trace in Fig. 1*B* is an intracellular recording from the LP neuron, with its sequential inhibition from the pyloric (PY), pyloric dilator (PD), and anterior burster (AB) neurons (note that AB and PD slow waves and spikes are synchronized), followed by the rebound burst in the LP neuron. The timing of the inhibitory inputs is shown by the extracellular recordings from the lvn (lateral ventricular nerve, Fig. 1*B*, bottom). The LP neuron somatic action potentials that ride on the top of the slow wave are highly attenuated by the LP neuron’s cable, as they are generated in the distant axon.

Fig. 1*C* shows the model structure and the composition of ion channels in each compartment (details in *Materials and Methods*). The soma-primary neurite compartment has a leak current (I_leak_), a modulator current (I_MI_), three potassium currents (I_Kd_, delayed-rectifier K^+^ current; I_A_, A-type K^+^ current; I_KCa_, calcium-activated K^+^ current), a calcium current (I_Ca_), and a hyperpolarization-activated inward current (I_h_). In addition to these currents, three inhibitory synaptic currents, from the AB, PD, and PY neurons (synAB, synPD, and synPY), are also distributed in the secondary and tertiary neurites. The axon has a I_leak_, I_Kd_, I_A_, and a voltage-gated Na^+^ current (I_Na_). A characteristic waveform from the LP neuron models is seen in Fig. 1*D*. The top trace is the model voltage in the soma-primary neurite compartment, while the bottom trace is the model voltage in the axon. The axon generates overshooting action potentials, and only a very small slow wave is visible, while the soma-primary neurite compartment replicates the somatic recordings seen in the biological neurons (Fig. 1*B*).

### Neuronal Robustness to Channel Conductance Variations.

**Fig. 2.**
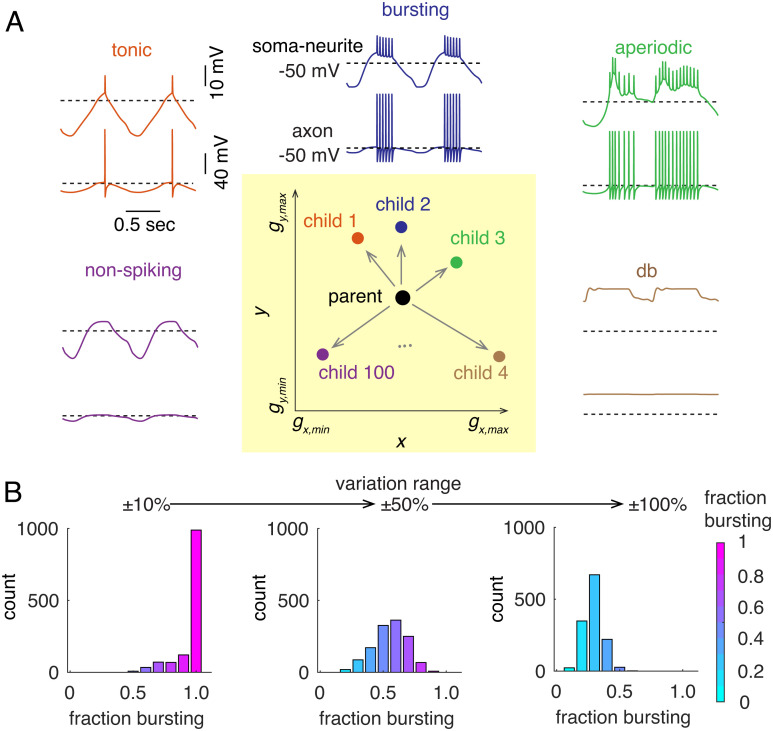
“Noise” perturbation protocol and neuronal resilience to conductance variations. (*A*) 2D schematic of the channel conductance perturbation protocol (*Bottom Middle*, shaded in yellow). *x* and *y* represent different types of ion channels. In each trial, all channel conductances of a parent model were simultaneously varied by random values (details in *Materials and Methods*). Each parent model (n = 1,292) generated new m child models after m-trial perturbations. Example firing patterns in child models include tonic spiking (child 1), bursting (child 2), aperiodic spiking (child 3), depolarization block (db, child 4), and non-spiking (child 100). (*B*) Histogram of the ratio of child models that are rebound bursters for each parent model, when models undergo different ranges of random channel conductance variations, ±10%, ±50%, and ±100% from left to right.

We used Taylor’s LP neuron models (5) as “parent” models (n = 1,292). In each parent model, we varied all of the 14 channel conductances simultaneously by different random values, based on their range of values in the 1,292 models. For each parent model, multiple runs (100 trial runs in initial simulations) with different sets of channel perturbations generated a number of “child” models, which displayed a variety of patterns of activity (Fig. 2*A*; *Materials and Methods*).

The child models showed a variety of behaviors, which can be generally classified as tonic spiking, rebound bursting, aperiodic spiking, depolarization block, or non-spiking (Fig. 2*A*). The simulation results of 100 trials for each parent model are summarized in Fig. 2*B*. For each parent model, the ratio of child models that are also rebound bursters with at least two spikes/burst was calculated to capture its robustness to channel conductance variations. A higher ratio suggests the parent model is more robust to channel conductance variations. When channel conductances were simultaneously varied within ±10% of their corresponding parameter ranges, the rebound bursting pattern was remarkably robust to the conductance variations. For 989 of the parent models, over 90% of the child models generated rebound bursting, and for 649 of the parent models, all the child models reliably generated rebound bursting. The overall high ratios of child models being rebound bursters indicate that the pattern of rebound bursting is relatively insensitive to the conductance variations in these parent models. When channel conductances were varied in larger ranges, more and more parent models showed decreased ratios of child models being rebound bursters, as reflected by their left-shifted distribution in the histograms (Fig. 2*B*).

It is interesting to estimate the size and structure of the 14-dimension manifold in which these models exist. Previous work on similar conductance-based models argued that models with similar behavior are in connected regions of parameter space (3, 7). For many parent models in these previous simulations (n = 649), the perturbation-generated child models reliably generate rebound bursting, suggesting that there are reasonably sized areas around those parent models with relatively consistent behaviors. We estimate that the size of the manifold in each channel conductance direction is at least 20% of their corresponding conductance ranges.

**Fig. 3.**
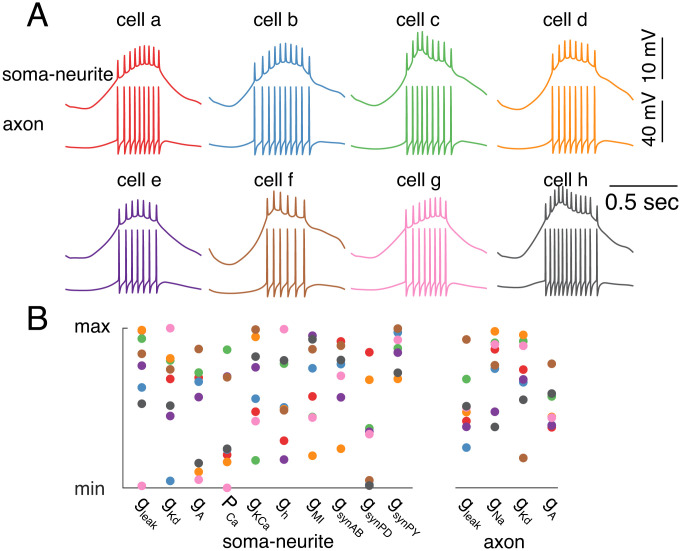
Robust models are widely distributed in parameter space. (*A*) The spike waveforms in the soma-primary neurite (*Top*) and axon (*Bottom*) of eight example parent models (models a–h), whose child models reliably produce rebound bursting after 10,000-trial perturbations when the variation range is ±10%. (*B*) The parameter distribution of these eight example models. All conductances were normalized to their corresponding conductance ranges in the whole model database, because their magnitudes are on different scales. Colors code different models (models a–h from panel *A*).

Therefore, we selected 37 elite parent models whose child models reliably generate rebound bursting for 100 trials of perturbations and have 3 to 11 spikes per burst when the variation range is ±10%. Then, we challenged each of them with 10,000 trials of perturbations. For 23 models, all their child models reliably generated rebound bursting, and in the other 14 models, the lowest ratio of the child models generating rebound bursting was 98.95%. These results again support the interpretation that this bursting behavior is relatively insensitive to random channel conductance variations. We plotted the spike waveforms and channel conductances for eight of the 23 models in Fig. 3. Except for larger values of the PY neuron synaptic input, the other channel conductance values spread broadly within their corresponding conductance ranges, suggesting that robust models exist widely in the 14-dimension parameter space.

**Fig. 4.**
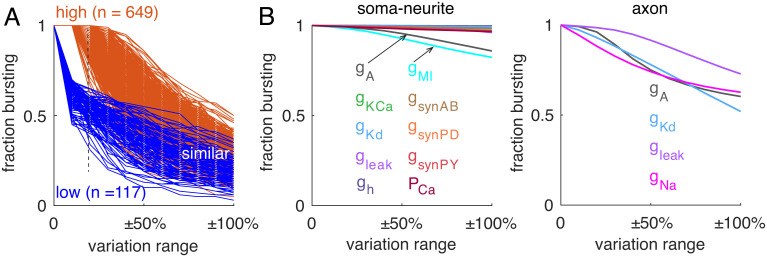
Factors regulating neuronal robustness. (*A*) The effect of variation range on neuronal resilience between two groups of neurons. They are grouped by the ratio of child models that are rebound bursters when the variation range is ±10% (orange group, 100%; blue group, <70%). (*B*) The sensitivity of neuronal resilience to individual channel conductance variations (coded by colors). *Left* and *Right* plots show the effect of varying one of the channel conductances in the soma-neurite and the axon, respectively. The *x*-axis represents conductance variation range, and the *y*-axis represents the average ratio of child models that are rebound bursters.

If one group of neurons shows an advantage in maintaining rebound bursting relative to another group within a small variation range, is this advantage preserved with larger variations? When the variation range increases, the two groups of neurons have similar ratios of maintaining the rebound bursting pattern regardless of their ratios with smaller variation ranges (Fig. 4*A*).

We also explored the effect of varying individual channel conductances on the robustness of rebound bursting. Compared with the effect of varying 14 channel conductances simultaneously, the models are more resilient to single channel conductance variations. In the soma and neurites, the model’s performance shows modest sensitivity to I_A_ and I_MI_ conductances. In contrast, varying each of the conductances in the axon changes the model’s firing pattern more significantly.

### Axonal Coupling with Other Compartments Determines Axonal Spiking during Bursts.

**Fig. 5.**
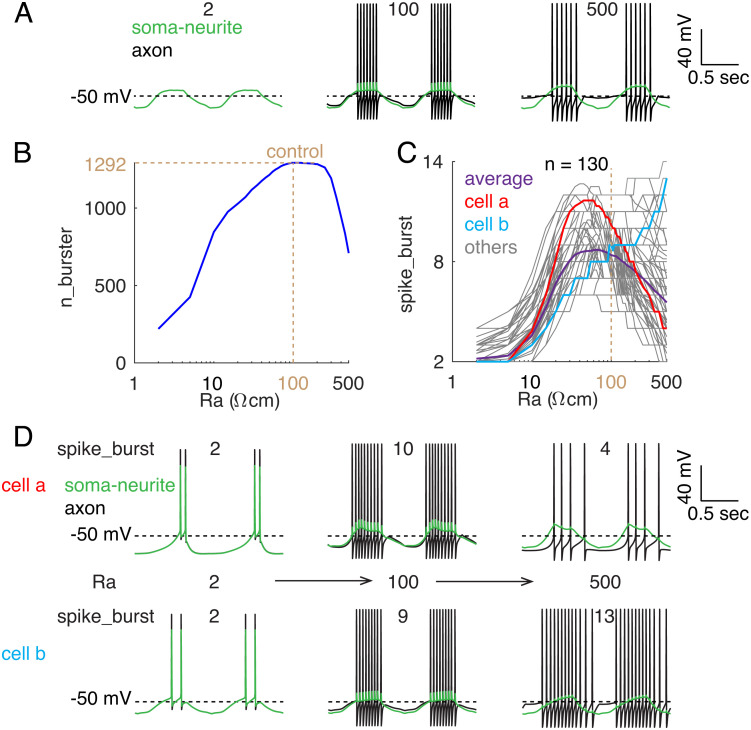
Effect of axonal coupling with other compartments. (*A*) Example spike waveforms of soma-neurite (green) and axon (black) when axonal Ra was changed from 100 Ωcm (*Middle*) to 2 Ωcm (*Left*) and 500 Ωcm (*Right*). (*B*) The relationship between axonal Ra and the bursting neuron population size (n_burster). n = 1,292 when Ra = 100 Ωcm under control condition. The *x*-axis is a logarithmic scale. (*C*) The relationship between axonal Ra and the number of spikes per burst (spike_burst) in neurons that generate rebound bursting regardless of Ra changes (n = 130). Purple, population average; red and cyan, two example neurons; gray, other neurons. The *x*-axis is a logarithmic scale. (*D*) The effect of axonal Ra on bursting changes of soma-neurite (green) and axon (black) in the example cells (cells a and b) highlighted in *C* (axonal Ra, 2 Ωcm → 500 Ωcm from left to right). In all simulations, there are no channel conductance variations and only axonal Ra was changed (equivalent to changing r2 in Fig. 1).

In the LP neuron, the soma and neurites generate rebound slow waves that evoke bursts of Na^+^ spikes in the axon. Therefore, axonal coupling with the soma and neurite sections is key to regulating the bursts of spikes. In the whole model database (n = 1,292), r2 was varied (by varying axonal axial resistivity Ra in NEURON, See *Materials and Methods*) to change the degree of axonal coupling with the remaining compartments, while other resistors were fixed to minimize the other potential changes, such as the input resistance at neurites. When the axonal Ra was changed, some models no longer produced rebound bursts (Fig. 5*A*). All models generated rebound bursting when the axonal Ra was in the range of 95 Ωcm to 125 Ωcm (Fig. 5*B*). For axonal Ra in the range of 40 Ωcm to 300 Ωcm, 1,175 (91%) models generated rebound bursting. These results suggest that the rebound bursting pattern is resilient to axonal Ra changes around the control value of 100 Ωcm. When axonal Ra deviated considerably from the control value, the number of rebound bursting neurons decreased. The models that generate rebound bursting with larger axonal Ra deviations are usually rebound bursters when the axonal Ra deviation is lower. For example, in the range of 2 Ωcm to 100 Ωcm, if a model is a rebound burster with a smaller axonal Ra, it is always a rebound burster when the axonal Ra is larger, with rare exceptions for the Ra from 15 Ωcm to 20 Ωcm (two exceptions) and from 35 Ωcm to 40 Ωcm (one exception).

The simulation results above show that the degree of coupling between the axon and the other compartments affects the population size of rebound bursting neurons. We selected 130 models that always generate rebound bursting for axonal Ra within 2 Ωcm to 500 Ωcm. The number of spikes per burst reflects the ability of the axon to spike during bursts. For most of these neurons, the number of spikes per burst shows biphasic changes with axonal Ra, as also reflected by the average changes (Fig. 5*C*). However, in several neuron models, this number monotonically increases within the tested Ra range (see example cell b in Fig. 5*C*).

When axonal Ra is small, the axon and other compartments are almost isopotential, as revealed by their nearly overlapped membrane potentials (Fig. 5*D*, *Left*). When they are strongly coupled, this reduces the ability of the axon to spike during bursts for two reasons. First, the axonal membrane potential after each spike is closer to the slow-wave peak (bottom Vm during the bursts), which reduces the recovery of Na^+^ channels from inactivation. Second, the other compartments exert a larger capacitance load on the axon during spiking (top Vm during the bursts). Therefore, when axonal Ra was 2 Ωcm, the number of spikes per burst was low. Increasing axonal Ra to make the axon more isolated from the other compartments should increase the axonal ability to spike during bursts, considering just the above two factors. However, the more isolated axon also reduces the axial current from the slow wave from the soma and neurites that triggers axonal spiking during bursts. Consequently, changes in the number of spikes per burst depend on the balance of these three factors when axonal Ra changes (Fig. 5*D*).

### Axonal Coupling with Other Compartments Determines Neuronal Robustness.

**Fig. 6.**
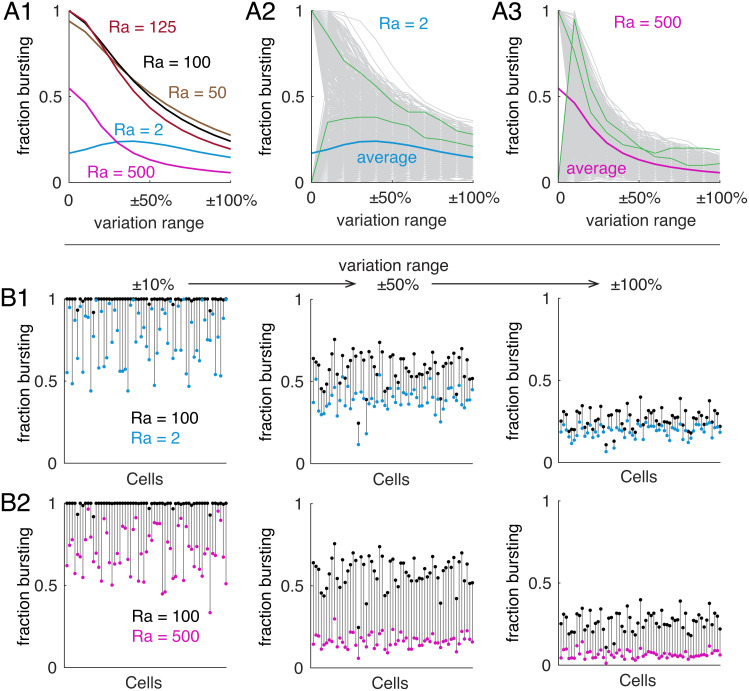
Effect of axonal coupling with other compartments on neuronal resilience to conductance variations. (*A*) The effect of axonal Ra on neuronal resilience to conductance variations for all the parent models. *A*1, the average ratio of child models that are rebound bursters with different axonal Ra values (coded by colors). *A*2 and *A*3 show individual neuronal ratios of child models that are rebound bursters for Ra = 2 Ωcm and 500 Ωcm, respectively. Green traces show typical example plots. Blue and magenta traces show population average ratios, as in *A*1. Gray traces show other individual neurons. (*B*) The comparison of each neuronal ratio of child models that are rebound bursters when axonal Ra is changed from the control value of 100 Ωcm. *B*1, Ra is changed from 100 Ωcm (black dots) to 2 Ωcm (blue dots); *B*2, Ra is changed from 100 Ωcm (black dots) to 500 Ωcm (magenta dots). From left to right, conductance variation range increases from ±10 to ±100%. Eighty of the total 131 elite parent models are shown. In all simulations, only axonal Ra was changed (equivalent to changing r2 in Fig. 1).

We explored how the axonal coupling with the other compartments affects neuronal robustness for all of the 1,292 parent models. When the axonal Ra is near the control value of 100 Ωcm, the average ratio of child models that are rebound bursters is insensitive to axonal Ra changes irrespective of conductance variation ranges (Fig. 6*A*1, Ra = 50, 100, and 125 Ωcm). However, when the coupling gets too strong (Ra = 2 Ωcm) or too weak (Ra = 500 Ωcm), the average ratio of child models that are rebound bursters is reduced. Under these conditions, for many parent models that lose rebound bursting, their child models become rebound bursters when the channel conductances are perturbed to appropriate ranges (Fig. 6 *A*2 and *A*3). When axonal Ra is 2 Ωcm, the average ratio of child models that are rebound bursters shows biphasic changes. Under these conditions, some of the original parent models are not rebound bursting neurons, but in the neighboring parameter space they may remain bursting (Fig. 6 *A*2 and A3). Fig. 6 suggests that the probability of finding rebound bursting neuron models near parent models is nearly unchanged when axonal Ra is near 100 Ωcm but decreases when Ra deviates further from its control value.

To explore how the axonal coupling with other compartments affects the ability of individual neurons to maintain rebound bursting when faced with channel conductance variations, we selected 131 elite models from the 1,292 parent models. These 131 elite parent models reliably generate rebound bursting when the axonal Ra is 2 Ωcm, 100 Ωcm, and 500 Ωcm. Strikingly, for each of these parent models, the ratio of child models that are rebound bursters consistently decreases when axonal Ra is changed from 100 Ωcm to 2 Ωcm and from 100 Ωcm to 500 Ωcm (Fig. 6*B*, only 80 cells were plotted, but all cells show the same trend), regardless of the conductance variation ranges. These simulation results suggest that the degree of axonal coupling with the other compartments determines the size of the manifold that corresponds to rebound bursting neurons and therefore the neuronal robustness to channel conductance variations.

## Discussion

Most neurons have complex structures over which ion channel distributions must be established such that the neuron’s characteristic electrical properties are maintained, given the inevitable noise that must accompany the ongoing turnover of channels in the membrane. It is known that some neurons display specific patterns of channel distributions that are thought to aid in the neuronal computations that these neurons perform (28–30). Therefore, it becomes important to determine the extent to which the spatial structure of neurons makes them more or less sensitive to fluctuations in channel densities across their structures.

In this work, we used a population of LP neuron compartmental models developed previously (5) to explore neuronal robustness to channel conductance variations. All members of this population met a series of criteria that ensure that they capture many of the essential features of the biological neurons whose behavior they were intended to replicate (5). At the same time, these models show considerable degeneracy in that the specific values of their conductances are highly variable (5). These models are more biologically “realistic” than single-compartment, point, neurons in many aspects (31), but they are not intended to replicate all of the detailed structures of individual STG neurons. The most critical property for this work is the separation of the spike initiation region from the remaining regions that are nearly electrotonically compact for slow waves (24).

Parameter sensitivity is universally sloppy in biology models, i.e., model outputs are sensitive to the variation of some parameters but not others (32). This “sloppiness” endows cellular resilience to corresponding parameter perturbations. In models of one or two dimensions, it is easy to find the parameter sets that define the boundary between different types of neuronal activity patterns (33, 34). However, as the number of dimensions increases, it becomes more challenging to characterize the parameter space for neurons with specific properties (7, 18). Although it may be possible using dimensional reduction or replotting to visualize high-dimensional parameter spaces (7, 35), it would be a daunting task to locate all possible rebound bursting models with a grid search for the 14-dimension parameter space here. It is impossible to know whether a sampling step size is small enough (3). Instead, we used random parameter perturbations. This approach does not provide an unambiguous map of the entire manifold. Nonetheless, if all child models are rebound bursters after exerting random and simultaneous conductance perturbations for sufficiently many trials, it argues the existence of a multi-dimension manifold that contains the very large majority of models of similar behavior (7). We did this in a subset of neuron models for 10,000 trials, and most of them reliably generated rebound bursting after perturbations (Fig. 3).

Multiple mechanisms likely exist that can allow neurons to compensate for perturbation-caused changes. In homeostatic models of intrinsic excitability and regulation of synaptic strength, both ion channel conductances and synaptic strength can be altered to recover neuronal and circuit function properties in response to deviations from a set point (16, 36–41). Some homeostatic mechanisms require new protein synthesis, and therefore, recovery may be slow and require hours or even days, whereas other processes may be more rapid (38, 40, 42, 43).

Neurons constantly face diverse perturbations such as temperature changes, neuromodulation, and stochastic changes in protein synthesis, but they rarely fail in healthy animals under physiological conditions. This argues that most neurons are not sitting too close to bifurcations, but neurons and circuits can tolerate some changes in their parameters without loss of function. The effect of morphology on the stability of physiological activity has received less attention than it probably merits. Nonetheless, there are examples of structural alterations that can be homeostatically regulated to control activity (44).

In many neurons, including those of the STG, neuronal Na^+^ spikes are initiated in the axon. In STG neurons, the slow waves that underlie bursting depend on channels in the soma and major neurites. Synaptic inputs and outputs occur on the processes, and these various regions of the neuron are coupled appropriately (5). Biological ranges of coupling can increase the density and the size of the manifolds that allow rebound bursting (Figs. 5 and 6). Recent data argue that the detailed morphology of STG neurons is not important for their overall function (23–25). Thus, instead of requiring the detailed specification of the number and location of each ion channel, this strategy may require only the specification of a generic structure that in turn depends on an appropriate range of values of their electrical coupling. Because of the simplified morphology used here, we cannot test whether more complex morphological structures can also enhance neuronal robustness, but this will be interesting to explore in the future. The control value of 100 Ωcm for the Ra in this model falls in the middle range of experimentally reported values (45) and was originally chosen to make the voltage waveforms of the LP neuron model roughly match experimental data (5). While individual LP and other STG neurons show animal-to-animal variability in channel conductances and channel mRNAs (1, 46–51), it is important to remember that the motor patterns produced by these neurons in the pyloric circuit are tightly maintained, and the LP neuron’s rebound burst is critical for its proper function (26). For this reason, we focused on the maintenance of rebound bursting to measure neuronal robustness. Apart from rebound bursting, other correlated properties also depend on the coupling (or separation) between the axon and the other compartments, such as somatic slow-wave amplitude. If all these properties are considered, the child neuron models that meet the selection criteria will be likely reduced (Figs. 5 and 6).

In crab STG neurons, although the morphological structures of these neurons exhibit significant cell-to-cell variations, they are electrotonically compact with gradually tapering neurites from the soma to the distal tips. This elegant, but not too rigid, solution makes graded transmission resilient to electrotonic decrement across sparsely distributed, synchronous presynaptic sites. Previously, we argued that the separation of synaptic integration and slow waves from axonal spike initiation zones may be a morphological strategy established to avoid shunting of synaptic currents (23–25, 52). Here, we use models to demonstrate that this division of labor can make neurons more robust to perturbations.

The findings in this study may be relevant to other neuronal systems. Spike bursts are widely observed in the cerebellum (53, 54), the neocortex (55), and the hippocampus (56). For example, the inferior olive neurons in the cerebellum show similarities to the LP neuron. Inferior olive neurons show subthreshold oscillations in the soma and then trigger axonal spike bursts to guide cerebellum-related movement and learning (53). Additionally, soma-axon coupling is also critical for the initiation of axonal spike bursts (complex spikes) in cerebellar Purkinje cells (17, 57). Because of the functional importance of these signals in cerebellar coding and learning, the reliability of triggering axonal bursting determines the robustness of corresponding behavioral functions. Moreover, neurons may utilize their entire structure to maintain a robust functional output in other spiking patterns (58, 59).

## Materials and Methods

We used the LP neuron multi-compartment models constructed previously (5) and implemented them in NEURON 7.6 (60). The LP neuron is a unipolar neuron (Fig. 1*C*). There is a thick primary neurite connecting the soma and the axon. A number of secondary neurites subsequently extend from the primary neurite, and more tertiary neurites ramify further from the secondary neurites. Accordingly, four compartments were used to approximate the axon initial segment (C = 0.039 nF), the soma-primary neurite (C = 0.8 nF), secondary neurites (C = 3.74 nF), and tertiary neurites (C = 5.50 nF). In Fig. 1, r1 = 2.50 × 10^5^ Ω; r2 = 1.80 × 10^7^ Ω; r3 = 1.13 × 10^6^ Ω; r4 = 2.26 × 10^7^ Ω. The axonal axial resistivity Ra corresponds to 100 Ωcm under the control condition (assume axon has a L = 354.5 µm, diam = 3.5 µm), in the middle range of experimental measurements. These models were constructed to replicate the rebound bursting properties observed in the LP neuron of the crab STG. We adopted 1,292 out of the original 1,304 cell models. The excluded cell models either need a long time to generate rebound bursting or didn’t generate this pattern after reaching a steady state, possibly because of NEURON version changes.

The current types and compartmental distributions are shown in Fig. 1. The detailed mathematical description of these currents can be found in the original work (5). All current models were modeled as ohmic currents, except I_Ca_. For the ohmic current, $I=\bar{g}*w*\left(V-E_{rev}\right)$ , $\overset{-}{g}$ is the maximal conductance, w is the product of activation and inactivation gate state variables, V is the transmembrane potential, and $E_{rev}$ is the reversal potential. The calcium current was modeled by Goldman–Hodgkin–Katz equations, $I=\bar{P_{\mathrm{Ca}}}*d_{\mathrm{Ca}}$ , where $d_{Ca}=$ $\begin{array}{c} {m_{\mathrm{Ca}}}^{3}*h_{\mathrm{Ca}}*N_{A}*q_{e}*z_{\mathrm{Ca}}*([{\mathrm{Ca}}^{2+}{]}_{i}*(-\xi )/(\exp(-\xi )-1)-[{\mathrm{Ca}}^{2+}{]}_{o}*\xi /(\exp(\xi )-1)) \end{array}$$\begin{array}{c} \;,\;\xi =z_{Ca}*q_{e}/\left(k*T\right)*V \end{array}$$\begin{array}{c} {m_{Ca}}^{3}*h_{Ca}*N_{A}*q_{e}*z_{\;Ca}*\;({\left[{Ca}^{2+}\right]}_{i}*-\xi /\;\left(\exp(-\xi )-1\right)-\;{\left[{Ca}^{2+}\right]}_{o}*\xi \;/ \end{array}$  $\begin{array}{c} \left(\exp\left(\xi \right)-1\right))\;,\;\xi =z_{Ca}*q_{e}/\left(k*T\right)*V\; \left(\exp\left(\xi \right)-1\right))\;,\;\xi =z_{Ca}*q_{e}/\left(k*T\right)*V \end{array}$ is the maximal permeability of the calcium ions, is the calcium channel activation gate, $\bar{P_{\mathrm{Ca}}}$ is the calcium channel inactivation gate, $m_{Ca}$ is the Avogadro’s constant, $h_{Ca}$ is the elementary charge, $N_{A}$ is the valence of a calcium ion, $q_{e}$ and $z_{Ca}$ are intracellular and extracellular calcium concentrations, ${\left[{Ca}^{2+}\right]}_{i}$ is the Boltzmann’s constant, and ${\left[{Ca}^{2+}\right]}_{o}$ is the Kelvin temperature. The three types of inhibitory synaptic inputs were approximated by fixed rhythmic patterns of synaptic conductances. For each type of synapse, the synaptic conductance was approximated by k , where T is the maximal synaptic conductance, $\begin{array}{c} g_{syn}=\bar{g_{\mathrm{syn}}}*s,\;\tau_{syn}*\dot{s}=s_{\infty }*(\left(V_{pre}-V_{thresh}\right)/v_{scale}) \end{array}$ is the fraction of synaptic activation, $\bar{g_{\mathrm{syn}}}$ is the time constant, s is the presynaptic voltage (smoothed experimental recordings), $\tau_{syn}$ is the synaptic threshold, and $V_{pre}$ is the voltage sensitivity of the synaptic activation. The AB neuron and PY neuron synapses have instantaneous dynamics and a reversal potential of −70 mV. The PD neuron synapse has slower dynamics (with a fixed time constant of 50 ms) and a reversal potential of −80 mV, in keeping with the known properties of these synapses (61).

For conductance perturbation simulations within a variation range, all 14 channel conductances were simultaneously and independently varied by values randomly sampled in the range of [−1, +1] * variation_range * (g*_x,max_*−g*_x,min_*). *x* represents a type of ionic current; g*_x,max_* and g*_x,min_* represent the maximal and the minimal conductances of current *x* in the parameter space, respectively. A conductance was set to zero if the value became negative after a random perturbation. A child model was classified as a rebound burster if it fires periodically, and there were at least two spikes per burst. For the elite parent models in Fig. 3, the first criterion is all their child models need to be rebound bursters when the variation range is ±10%. The second criterion is the number of spikes per burst in the parent models and their corresponding child models need to be in the range of 3 to 11 (5). For the results in Figs. 5 and 6, by varying axonal Ra, r2 was varied to simulate its different degrees of coupling to the other compartments of the neuron. We constrained the r2 in the range of 3.60 × 10^5^ to 9 × 10^7^ Ω (axonal Ra in the range of 2 to 500 Ωcm) to maintain a sufficient number of neurons in Figs. 5*C* and 6*B* to compare the changes in individual neurons.

## Data Availability

Model code is available in ModelDB (accession number: 267621).
